# *N*,*N*′-Bis(2,2,3,3,4,4,4-hepta­fluoro­butyl)naphthalene-1,4:5,8-tetra­carboximide

**DOI:** 10.1107/S1600536808036738

**Published:** 2008-11-13

**Authors:** Deepak Shukla, Manju Rajeswaran, Wendy G. Ahearn, Dianne M. Meyer

**Affiliations:** a Eastman Kodak Company, Rochester, NY 14650-2106, USA

## Abstract

The title mol­ecule, C_22_H_8_F_14_N_2_O_4_, lies across a crystallographic inversion center with the naphthalene diimide core essentially planar (mean deviation from plane is 0.0583 Å). The CF_2_ groups in the perfluorobutyl chains are in an energetically favorable all *trans* conformation. In the crystal structure, mol­ecules are packed in slightly displaced layers so that the side chains overlap the aromatic naphthalene diimide rings, thus minimizing any possible π–π overlap.

## Related literature

For general background on the semic-conducting properties and use of this class of materials in organic thin-film transistor applications, see: Chesterfield *et al.* (2004*a* ▶,*b* ▶); Facceti *et al.* (2008 ▶); Jones *et al.* (2004 ▶); Katz *et al.* (2000*a* ▶,*b* ▶); Kazmaier & Hoffmann (1994 ▶); Klebe *et al.* (1989 ▶); Shukla *et al.* (2008 ▶); Wurthner (2004 ▶).
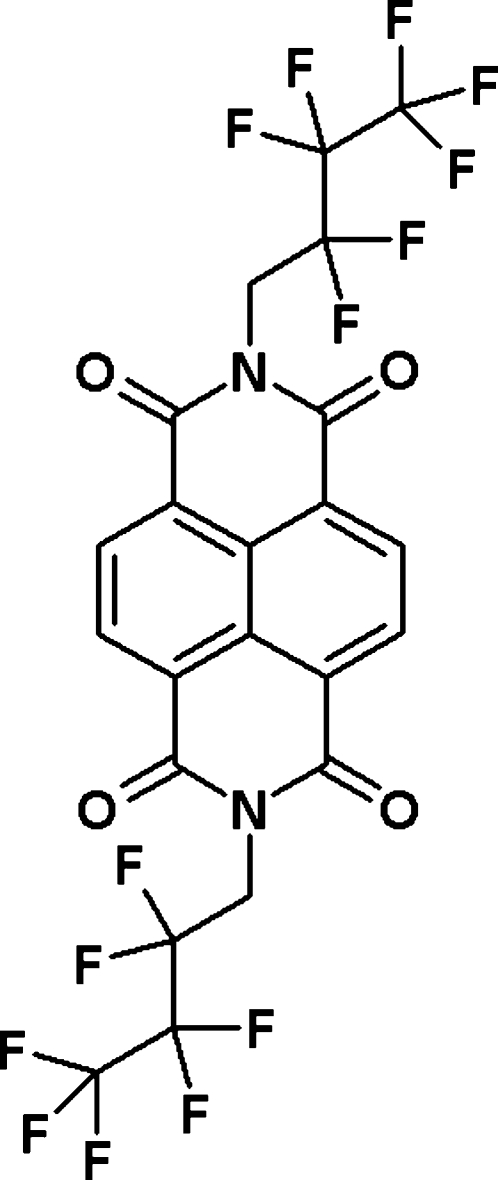


## Experimental

### 

#### Crystal data

C_22_H_8_F_14_N_2_O_4_
*M* *_r_* = 630.30
Triclinic, 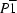

*a* = 5.1910 (5) Å
*b* = 10.1459 (12) Å
*c* = 11.5988 (15) Å
α = 66.693 (4)°
β = 79.064 (4)°
γ = 89.115 (7)°
*V* = 549.64 (11) Å^3^
*Z* = 1
Mo *K*α radiation
μ = 0.21 mm^−1^
*T* = 293 (2) K
0.15 × 0.10 × 0.05 mm

#### Data collection

Nonius KappaCCD diffractometer
Absorption correction: none
3049 measured reflections
2094 independent reflections
909 reflections with *I* > 2σ(*I*)
*R*_int_ = 0.057

#### Refinement

*R*[*F* ^2^ > 2σ(*F* ^2^)] = 0.067
*wR*(*F* ^2^) = 0.223
*S* = 0.93
2094 reflections
190 parameters
H-atom parameters constrained
Δρ_max_ = 0.23 e Å^−3^
Δρ_min_ = −0.23 e Å^−3^

### 

Data collection: *COLLECT* (Nonius, 2000 ▶); cell refinement: *SCALEPACK* (Otwinowski & Minor, 1997 ▶); data reduction: *DENZO* (Otwinowski & Minor, 1997 ▶) and *SCALEPACK*; program(s) used to solve structure: *SHELXTL* (Sheldrick, 2008 ▶); program(s) used to refine structure: *SHELXTL*; molecular graphics: *SHELXTL* and *Materials Studio* (Accelrys, 2002 ▶); software used to prepare material for publication: *publCIF* (Westrip, 2008 ▶).

## Supplementary Material

Crystal structure: contains datablocks I, global. DOI: 10.1107/S1600536808036738/lh2728sup1.cif

Structure factors: contains datablocks I. DOI: 10.1107/S1600536808036738/lh2728Isup2.hkl

Additional supplementary materials:  crystallographic information; 3D view; checkCIF report

## Figures and Tables

**Fig. 1.**
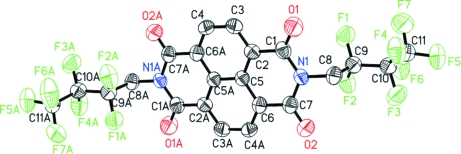
Molecular structure of the title compound, with the atom numbering scheme. Displacement ellipsoids are drawn at the 50% probability level and H atoms are omitted for clarity.

**Fig. 2.**
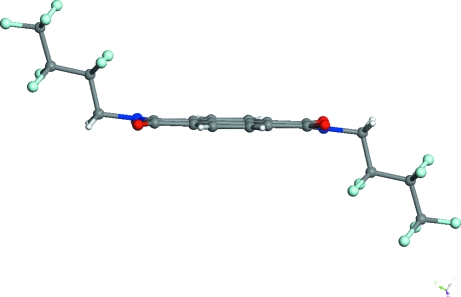
A diagram illustrating planar naphthalene diimide core and trans configuration of perfluorobutyl chains on diiimide N atoms.

**Fig. 3.**
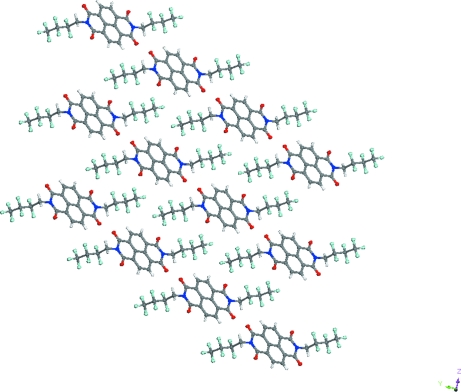
Unit cell packing showing layered structure.

## supplementary crystallographic information

### Comment

Amongst n-type semiconductors used in organic thin film transistors, perylene diimides (PDIs) and naphthalene diimides (NDIs) have attracted considerable attention. The π -orbital wavefunctions in these systems form nodes at the two nitrogen positions in the imide rings. Indeed, it has been shown that semiconducting properties and device performance of these materials is very sensitive to the nature of substituents on the diimide nitrogen atoms. The title compound *N*,*N*'-Bis(1*H*,1*H*-perfluorobutyl) naphthalene- 1,4,5,8-tetracarboxylic acid diimide(I) has been shown to exhibit good n-type semiconducting behavior and OTFTs made incorporating I can be operated in air. The latter property has been ascribed to the denser packing of fluorinated alkyl chains in thin film.

Naphthalene diimide (NDI) and perylene diimide (PDI) based systems have been studied extensively (Chesterfield, *et al.*, 2004*a*; Chesterfield *et al.*, 2004*b*; Facceti *et al.*,  2008; Jones, *et al.*, 2004; Katz, *et al.*, 2000*a*; Katz, *et al.*, 2000*b*). We report here the structure of the title diimide molecule (I) (Fig. 1 and Fig 2). In the crystal structure, molecules are packed in slightly displaced layers so that the side chains overlap the aromatic naphthalene diimide rings, thus resulting in minimizing any possible π-π overlap (Fig .3).

### Experimental

The method described in Katz *et al.*, 2000*a*, was followed for preparation of the title compound (I). Crystals of title (I) appeared during powder X-ray diffraction data collection of the dry lot sample. The crystals were weakly diffracting, but we were unable to get better quality crystals. Diffraction data were collected on various crystals, and the results of structure determination using best data set results are reported here.

### Refinement

All H-atoms were positioned geometrically and refined using a riding model with d(C—H) = 0.93 Å, *U*_iso_=1.2*U*_eq_ (C) for aromatic 0.97 Å, *U*_iso_ = 1.2*U*_eq_ (C) for CH_2_ atoms.

### Crystal data

**Table:**

C_22_H_8_F_14_N_2_O_4_	*Z* = 1
*M**_r_* = 630.30	*F*_000_ = 312
Triclinic, *P*1	*D*_x_ = 1.904 Mg m^−^^3^
Hall symbol: -P 1	Mo *K*α radiation λ = 0.71073 Å
*a* = 5.1910 (5) Å	Cell parameters from 4558 reflections
*b* = 10.1459 (12) Å	θ = 1.0–26.7º
*c* = 11.5988 (15) Å	µ = 0.21 mm^−^^1^
α = 66.693 (4)º	*T* = 293 (2) K
β = 79.064 (4)º	Needle, pink
γ = 89.115 (7)º	0.15 × 0.10 × 0.05 mm
*V* = 549.64 (11) Å^3^	

### Data collection

**Table:**

Nonius KappaCCD diffractometer	2094 independent reflections
Radiation source: fine-focus sealed tube	909 reflections with *I* > 2σ(*I*)
Monochromator: graphite	*R*_int_ = 0.057
Detector resolution: 9 pixels mm^-1^	θ_max_ = 26.6º
*T* = 293(2) K	θ_min_ = 4.1º
φ and ω scans	*h* = −6→6
Absorption correction: none	*k* = −12→11
3049 measured reflections	*l* = −14→12

### Refinement

**Table:**

Refinement on *F*^2^	Secondary atom site location: difference Fourier map
Least-squares matrix: full	Hydrogen site location: inferred from neighbouring sites
*R*[*F*^2^ > 2σ(*F*^2^)] = 0.067	H-atom parameters constrained
*wR*(*F*^2^) = 0.223	*w* = 1/[σ^2^(*F*_o_^2^) + (0.1*P*)^2^ + 0.3623*P*] where *P* = (*F*_o_^2^ + 2*F*_c_^2^)/3
*S* = 0.93	(Δ/σ)_max_ < 0.001
2094 reflections	Δρ_max_ = 0.23 e Å^−^^3^
190 parameters	Δρ_min_ = −0.23 e Å^−^^3^
Primary atom site location: structure-invariant direct methods	Extinction correction: none

### Special details

**Table:**

Geometry. All e.s.d.'s (except the e.s.d. in the dihedral angle between two l.s. planes) are estimated using the full covariance matrix. The cell e.s.d.'s are taken into account individually in the estimation of e.s.d.'s in distances, angles and torsion angles; correlations between e.s.d.'s in cell parameters are only used when they are defined by crystal symmetry. An approximate (isotropic) treatment of cell e.s.d.'s is used for estimating e.s.d.'s involving l.s. planes.
Refinement. Refinement of *F*^2^ against ALL reflections. The weighted *R*-factor *wR* and goodness of fit *S* are based on *F*^2^, conventional *R*-factors *R* are based on *F*, with *F* set to zero for negative *F*^2^. The threshold expression of *F*^2^ > σ(*F*^2^) is used only for calculating *R*-factors(gt) *etc*. and is not relevant to the choice of reflections for refinement. *R*-factors based on *F*^2^ are statistically about twice as large as those based on *F*, and *R*- factors based on ALL data will be even larger.

### Fractional atomic coordinates and isotropic or equivalent isotropic displacement parameters (Å^2^)

**Table:**

	*x*	*y*	*z*	*U*_iso_*/*U*_eq_	
F1	0.1050 (6)	0.4145 (3)	0.7561 (3)	0.0814 (11)	
F2	0.1713 (6)	0.5174 (3)	0.8788 (3)	0.0828 (12)	
F3	0.5849 (6)	0.3623 (4)	0.9393 (3)	0.0873 (12)	
F4	0.5572 (7)	0.2684 (3)	0.8049 (3)	0.0883 (12)	
F5	0.3622 (7)	0.0974 (4)	1.0537 (3)	0.0931 (12)	
F6	0.0806 (7)	0.2472 (4)	1.0654 (4)	0.1050 (15)	
F7	0.0800 (9)	0.1477 (4)	0.9346 (4)	0.1234 (17)	
O1	0.2493 (8)	0.6081 (4)	0.4924 (4)	0.0742 (12)	
O2	0.5194 (8)	0.8024 (4)	0.7439 (4)	0.0725 (12)	
N1	0.3566 (7)	0.6967 (4)	0.6298 (4)	0.0484 (11)	
C1	0.2325 (10)	0.7037 (5)	0.5308 (5)	0.0528 (14)	
C2	0.0799 (9)	0.8305 (5)	0.4790 (5)	0.0467 (12)	
C3	−0.0607 (10)	0.8401 (5)	0.3865 (5)	0.0562 (14)	
H3	−0.0557	0.7679	0.3559	0.067*	
C4	−0.2109 (10)	0.9584 (5)	0.3386 (5)	0.0541 (14)	
H4	−0.3058	0.9637	0.2767	0.065*	
C5	0.0752 (9)	0.9387 (5)	0.5243 (4)	0.0440 (12)	
C6	0.2199 (9)	0.9345 (5)	0.6184 (5)	0.0482 (13)	
C7	0.3779 (10)	0.8079 (5)	0.6691 (5)	0.0523 (14)	
C8	0.4892 (9)	0.5658 (5)	0.6912 (5)	0.0537 (14)	
H8A	0.6355	0.5891	0.7224	0.064*	
H8B	0.5580	0.5257	0.6292	0.064*	
C9	0.2958 (10)	0.4562 (6)	0.8023 (5)	0.0544 (14)	
C10	0.4188 (10)	0.3238 (5)	0.8827 (5)	0.0550 (14)	
C11	0.2281 (13)	0.2022 (6)	0.9871 (6)	0.0676 (16)	

### Atomic displacement parameters (Å^2^)

**Table:**

	*U*^11^	*U*^22^	*U*^33^	*U*^12^	*U*^13^	*U*^23^
F1	0.061 (2)	0.082 (2)	0.086 (2)	−0.0057 (16)	−0.0279 (18)	−0.0114 (19)
F2	0.091 (2)	0.063 (2)	0.074 (2)	0.0213 (17)	0.0167 (18)	−0.0222 (18)
F3	0.074 (2)	0.098 (3)	0.083 (3)	−0.0005 (18)	−0.0340 (18)	−0.021 (2)
F4	0.108 (3)	0.077 (2)	0.070 (2)	0.0396 (19)	−0.0012 (19)	−0.0278 (19)
F5	0.107 (3)	0.071 (2)	0.078 (2)	0.025 (2)	−0.018 (2)	−0.0064 (19)
F6	0.101 (3)	0.087 (3)	0.082 (3)	0.023 (2)	0.026 (2)	−0.007 (2)
F7	0.147 (4)	0.081 (3)	0.125 (4)	−0.039 (3)	−0.057 (3)	−0.008 (3)
O1	0.095 (3)	0.070 (3)	0.073 (3)	0.031 (2)	−0.024 (2)	−0.042 (2)
O2	0.079 (3)	0.067 (3)	0.080 (3)	0.0131 (19)	−0.038 (2)	−0.029 (2)
N1	0.054 (2)	0.040 (2)	0.051 (3)	0.0061 (18)	−0.010 (2)	−0.018 (2)
C1	0.059 (3)	0.046 (3)	0.052 (3)	0.009 (3)	−0.006 (3)	−0.021 (3)
C2	0.049 (3)	0.048 (3)	0.044 (3)	0.003 (2)	−0.002 (2)	−0.023 (3)
C3	0.068 (3)	0.051 (3)	0.057 (3)	0.006 (3)	−0.015 (3)	−0.028 (3)
C4	0.064 (3)	0.058 (3)	0.047 (3)	0.005 (3)	−0.016 (2)	−0.026 (3)
C5	0.045 (3)	0.044 (3)	0.038 (3)	0.001 (2)	−0.002 (2)	−0.014 (2)
C6	0.046 (3)	0.051 (3)	0.041 (3)	0.003 (2)	−0.003 (2)	−0.014 (3)
C7	0.051 (3)	0.052 (3)	0.044 (3)	0.000 (2)	−0.004 (3)	−0.012 (3)
C8	0.050 (3)	0.055 (3)	0.051 (3)	0.009 (2)	−0.010 (2)	−0.017 (3)
C9	0.053 (3)	0.056 (3)	0.057 (4)	0.010 (3)	−0.010 (3)	−0.026 (3)
C10	0.060 (3)	0.058 (3)	0.054 (3)	0.014 (3)	−0.011 (3)	−0.031 (3)
C11	0.085 (4)	0.056 (4)	0.058 (4)	0.003 (3)	−0.024 (4)	−0.015 (3)

### Geometric parameters (Å, °)

**Table:**

F1—C9	1.357 (6)	C2—C5	1.389 (6)
F2—C9	1.341 (6)	C3—C4	1.402 (7)
F3—C10	1.325 (6)	C3—H3	0.9300
F4—C10	1.338 (6)	C4—C6^i^	1.360 (7)
F5—C11	1.321 (6)	C4—H4	0.9300
F6—C11	1.294 (7)	C5—C6	1.425 (6)
F7—C11	1.314 (6)	C5—C5^i^	1.435 (9)
O1—C1	1.213 (6)	C6—C4^i^	1.360 (7)
O2—C7	1.223 (6)	C6—C7	1.491 (7)
N1—C7	1.387 (6)	C8—C9	1.522 (7)
N1—C1	1.398 (6)	C8—H8A	0.9700
N1—C8	1.467 (6)	C8—H8B	0.9700
C1—C2	1.477 (7)	C9—C10	1.513 (7)
C2—C3	1.380 (7)	C10—C11	1.539 (8)
			
C7—N1—C1	124.8 (4)	N1—C8—C9	109.7 (4)
C7—N1—C8	117.3 (5)	N1—C8—H8A	109.7
C1—N1—C8	117.8 (4)	C9—C8—H8A	109.7
O1—C1—N1	120.4 (5)	N1—C8—H8B	109.7
O1—C1—C2	123.0 (5)	C9—C8—H8B	109.7
N1—C1—C2	116.6 (5)	H8A—C8—H8B	108.2
C3—C2—C5	120.2 (5)	F2—C9—F1	105.5 (4)
C3—C2—C1	119.6 (5)	F2—C9—C10	108.6 (4)
C5—C2—C1	120.2 (5)	F1—C9—C10	108.7 (4)
C2—C3—C4	120.1 (5)	F2—C9—C8	110.1 (4)
C2—C3—H3	119.9	F1—C9—C8	109.2 (4)
C4—C3—H3	119.9	C10—C9—C8	114.3 (4)
C6^i^—C4—C3	120.9 (5)	F3—C10—F4	107.7 (4)
C6^i^—C4—H4	119.6	F3—C10—C9	109.0 (4)
C3—C4—H4	119.6	F4—C10—C9	108.5 (4)
C2—C5—C6	122.3 (5)	F3—C10—C11	107.7 (5)
C2—C5—C5^i^	120.6 (6)	F4—C10—C11	107.2 (5)
C6—C5—C5^i^	117.2 (6)	C9—C10—C11	116.4 (5)
C4^i^—C6—C5	121.0 (5)	F6—C11—F7	109.6 (6)
C4^i^—C6—C7	121.4 (5)	F6—C11—F5	108.2 (5)
C5—C6—C7	117.7 (5)	F7—C11—F5	107.3 (5)
O2—C7—N1	121.5 (5)	F6—C11—C10	111.6 (5)
O2—C7—C6	120.7 (5)	F7—C11—C10	110.2 (5)
N1—C7—C6	117.8 (5)	F5—C11—C10	109.8 (5)
			
C7—N1—C1—O1	171.2 (4)	C4^i^—C6—C7—N1	173.8 (4)
C8—N1—C1—O1	−4.5 (7)	C5—C6—C7—N1	−5.4 (6)
C7—N1—C1—C2	−9.8 (7)	C7—N1—C8—C9	95.1 (5)
C8—N1—C1—C2	174.5 (4)	C1—N1—C8—C9	−88.9 (5)
O1—C1—C2—C3	2.8 (8)	N1—C8—C9—F2	−50.4 (6)
N1—C1—C2—C3	−176.1 (4)	N1—C8—C9—F1	65.0 (6)
O1—C1—C2—C5	−178.0 (5)	N1—C8—C9—C10	−173.0 (5)
N1—C1—C2—C5	3.1 (7)	F2—C9—C10—F3	−59.0 (5)
C5—C2—C3—C4	−0.6 (7)	F1—C9—C10—F3	−173.3 (4)
C1—C2—C3—C4	178.6 (4)	C8—C9—C10—F3	64.4 (6)
C2—C3—C4—C6^i^	0.4 (8)	F2—C9—C10—F4	−176.0 (4)
C3—C2—C5—C6	−179.1 (4)	F1—C9—C10—F4	69.7 (5)
C1—C2—C5—C6	1.7 (7)	C8—C9—C10—F4	−52.6 (6)
C3—C2—C5—C5^i^	0.0 (8)	F2—C9—C10—C11	63.0 (6)
C1—C2—C5—C5^i^	−179.2 (5)	F1—C9—C10—C11	−51.3 (7)
C2—C5—C6—C4^i^	−179.8 (4)	C8—C9—C10—C11	−173.5 (5)
C5^i^—C5—C6—C4^i^	1.1 (8)	F3—C10—C11—F6	65.9 (6)
C2—C5—C6—C7	−0.6 (7)	F4—C10—C11—F6	−178.4 (5)
C5^i^—C5—C6—C7	−179.7 (5)	C9—C10—C11—F6	−56.8 (7)
C1—N1—C7—O2	−169.7 (5)	F3—C10—C11—F7	−172.1 (5)
C8—N1—C7—O2	6.0 (7)	F4—C10—C11—F7	−56.4 (6)
C1—N1—C7—C6	11.0 (7)	C9—C10—C11—F7	65.2 (7)
C8—N1—C7—C6	−173.3 (4)	F3—C10—C11—F5	−54.1 (7)
C4^i^—C6—C7—O2	−5.5 (7)	F4—C10—C11—F5	61.6 (6)
C5—C6—C7—O2	175.3 (4)	C9—C10—C11—F5	−176.8 (5)

Symmetry codes: (i) −*x*, −*y*+2, −*z*+1.
